# The Missing Memristor has Not been Found

**DOI:** 10.1038/srep11657

**Published:** 2015-06-25

**Authors:** Sascha Vongehr, Xiangkang Meng

**Affiliations:** 1 National Laboratory of Solid State Microstructures, College of Engineering and Applied Sciences, and Institute of Materials Engineering, Nanjing University, Jiangsu, China

## Abstract

In 1971, not only the theoretical and by definition already existing ‘ideal memristor’ concept was introduced, but a real memristor device was suggested on grounds of the already known real inductors. The latter is a scientifically significant hypothesis grounded in fundamental symmetries of basic physics, here electro-magnetism. 2008 claimed the discovery of the “missing memristor.” Controversy arose: The devices were not new, and the hypothesized device needs magnetism but has no material memory, while the available devices constitute analogue memory that would work in a world without magnetism. Nevertheless, even the originator of the prediction accepted the discovery. Defenders of the 2008 claim emphasize that the devices are not merely ‘memristive systems,’ which is however a distinction defined in 1976, not 1971. We clarify widely confused concepts and maintain that the originally hypothesized real memristor device is missing and likely impossible. The argument is illustrated also by finding an ideal *mechanical* memristor element and purely mechanical memristive systems, and hypothesizing a real mechanical memristor device that requires *inert mass* just like the 1971 implied device requires *magnetic induction*.

The ‘ideal resistor’ (or resistance *R*), ‘ideal capacitor’ *C*, and ‘ideal inductor’ *L* are the traditionally known *basic two-terminal circuit elements* (BCE). They were known because they have closely corresponding simple devices; for example two metal plates make a *real* (non-ideal) capacitor *device*; a metal coil is a real inductor device. It is crucial for the ideal BCE that they are independent of each other; further details on this are in the Supplementary Information (SI); and it is therefore that real devices are never ideal. For example a real resistor device has always some capacitance and inductance, too. On grounds of relevant symmetries, one can define a fourth BCE, ‘ideal memristor’ or ‘memristance’ *M*, and also hypothesize a real memristor device, as done in 1971^1^. The ideal memristor BCE is a theoretical concept that exists by definition, but a real memristor device was *missing*, and it was called the *missing fourth device* because the third, namely the real inductor device, was *not* missing; this is important for appreciating what kind of scientifically deeply significant thing is actually missing, how that real memristor device is thereby implicitly defined. The literature often calls all the different concepts here and below simply “memristor,” often in spite of being aware of crucial differences, which created much confusion. Regardless real or ideal, a ‘memristive system,’ which was defined only in 1976^2^, can be for our purposes sufficiently understood by focusing on charge-controlled systems: *M*_(*Q*)_ may depend directly only on charge *Q* (we call this “perfect” memristor to be consistent with the 2008 claim), while a ‘memristive system’ depends also on something else such as the current *I* for example, thus *M*_(*Q*,*I*)_. Notice that one could now think that an ideal/real/true/genuine/perfect memristor (all have been used in the literature) is one that can be described by *M*_(*Q*)_, not needing *M*_(*Q*,*I*)_. An alternative definition thereby emerged, namely “memristor” being tacitly (re)defined as meaning *not-just-memristive* (but perfect). The 1971 proposal did not know this later categorization, and the hypothesized missing real memristor device cannot be reduced to this, as we will explain in detail.

In 2008, the discovery of a “missing memristor” was announced with three (!) basically simultaneously timed, overlapping *Nature* group articles^3^,^4^,^5^ and on the front pages of major newspapers, all as if an almost 40 years ago predicted, deeply scientifically significant hypothesis had been finally proven. There was immediately controversy around that the devices are neither new nor the 1971 proposed real memristor device^6^,^7^. Similar devices were already discovered in 1995^8^, but those early discoverers do still not think that their devices are real memristors. Memristive behavior is known from thin films since even before 1971^9^. Before 2008, such devices and nonvolatile memory applications^10^ were correctly not called memristors^11^,^12^. The 2008 claim showed that the films of TiO_2_ between metals, well known since the 1960s, can be described as resistors with memory^13^, and “memristors” understood merely as nonlinear resistors with memory have been described by Kubo theory in the 1950s^14^. Novel in 2008 was merely the widely emphasized claim that such devices are *the* long sought “missing memristor,” but looking closer, it turns out that the authors were apparently missing something they call “perfect memristor,” the meaning of which accords to the mentioned tacit redefinition of “memristor” as *not-just-memristive*. It is highly doubtful that wide media attention would be given to such a mere technicality described around long known devices. Even if the existence of a perfect memristor was firstly recognized in 2008, the world is *missing* something else entirely: a real memristor device as suggested in 1971 on grounds of EM symmetry, namely on par with the known real inductor, the fourth next to that known third, *both* of them impossible without magnetic flux.

That the originator of the 1971 hypothesis defends the purported discovery^15^,^16^ submits the case as rich and unprecedented to the sociology of science. However, accepting the missing memristor as discovered hinders actually discovering it or recognizing that such is impossible. The issue is therefore directly relevant to the exact sciences also if one were to dismiss any relevance or involvement of ‘unscientific’ aspects or practices in how the scientific community socially constructs knowledge. The originally implied real memristor device stands as an important hypothesized scientific entity in need of verification or disproof. Sadly, many criticisms, including our own previous attempt,7 confuse the issue further by mixing in fundamentally unrelated problems. Rejecting that known devices are suddenly supposedly a breakthrough for artificial brains can be relevant for whether “hype” rather than honest confusion could be involved for example, but it says nothing about whether a real memristor device was found or can exist; this justifies our narrow focus.

Mendeleev’s 1870 prediction of chemical elements is cited as a relevant similarity in the 1971 proposal. Mendeleev’s hypothesis rested on empty cells in the periodic table of the elements^17^ and the memristor was similarly also a vacant cell in a two dimensional table (Fig. 1b and Fig. 2b). However, the involved symmetries are not this trivial, which we will discuss in detail. We thereby show that the memristor proposal is indeed comparable with for example Murray Gell-Mann noticing gaps in the patterns of SU(3) representations and thus predicting subatomic particles. Often, such proposals guide the discovery, much like Le Verrier’s 1846 prediction of Neptune’s existence and location, and successful historical predictions such as Dirac’s positron in 1928 were confirmed relatively soon. The claimed memristor discovery reminds of the claimed detection of gravitational waves by Joseph Weber in the late 1960s. Also initially accepted, it was discredited in the mid 1970s and led to a number of sociological analyses^18^,^19^. IBM physicist Richard Garwin built a similar detector but in half a year found only one pulse due to noise^19^. Physicist David Douglass showed that Weber’s computer program combined noise and artifices due to data binning, which resulted in daily coincidence signals^20^. The fatal rebuttal however, like ours here, focused on fundamental theory. (Garwin calculated that if Weber’s detection were real, the universe would have converted all its energy into gravitational radiation in only 50 million years.)

## Refusing the Discovery of the Originally Proposed Memristor Device

The difference between the ideal memristor and the hypothesized real memristor device is best understood by showing how all four ideal BCE arise in circuit theory *without* magnetism. This fact is often stated in defense of the 2008 claim, so we must show what this means and how it differs from circuit theory with magnetism.

### Starting circuit theory without magnetism, with or without Maxwell

**Figure 1:**
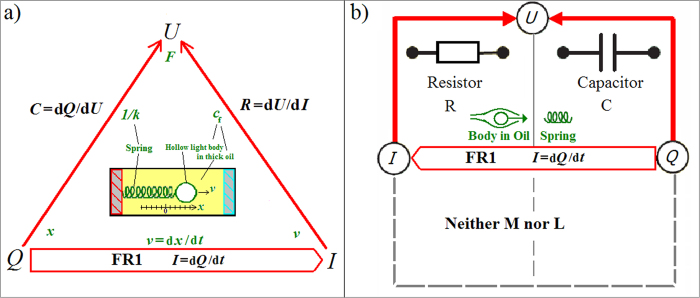
The symmetry of the three fundamental circuit variables *Q*, *I*, and *U*; the mechanical analog of a light hollow body moving in viscous oil is inset; its variables are shown in green: (**a**) Electrical force fields give rise to voltage *U*, which erects a triangle above the first fundamental relation FR1. The two new sides correspond to the two non-magnetic BCE. Charge dependent resistors *R*_(*Q*)_ correspond to position dependent drag coefficients *c*_f(*x*)_, e.g. due to differences in oil density maintained by a temperature gradient. (**b**) *M* and *L* are *both* absent in the more usual representation.

A circuit theory follows charges in a circuit. The *current-charge relation I* = d*Q*/d*t* defines the current *I* as a time derivative of the charge *Q*. This *first fundamental relation* (FR1) of circuit theory applies equally to mass flows, or to velocity *v* = d*x*/d*t* with the “charge” being *x*. Without force fields, there is only one arrow in Fig. 1a, namely FR1. Electrical charges have electrical force fields between them, and a voltage *U* arises. We can equally introduce force if FR1 is *v* = d*x*/d*t*, namely if a light hollow body is attached to a spring so that charge *x* is the spring displacement and *U* is *F* = −*x k* with Hooke’s spring stiffness *k*. All is submerged in thick viscous oil in an orbiting satellite (no gravity, no buoyancy), therefore *F* is coupled to friction force *F* = −*c*_f_*v* with drag coefficient *c*_f_^21^. The symmetry is now that of a triangle subtended by the three fundamental circuit variables *Q*, *I* and *U* (Fig. 1a). The (electrical) force field, via *U*, introduced two edges, *Q*-to-*U* and *I*-to-*U*. These allow two further binary relations apart from FR1. Those two correspond to the first two BCE, namely *C*_(*U*,*Q*)_ = d*Q*/d*U* and


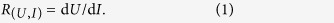


In the body/spring/oil (BSO) system, the spring “stores” charge *x*, *C* is the inverse of *k*, and *R* is energy dissipating drag-resistance (−*c*_f_ = *F*/*v*). The arrows in Fig. 1 indicate that physical charge is prior to the definition of current; the general force or tension term *U*, although physical and in some sense prior, is only discovered via the real capacitor or resistor devices and manifests itself conditioned by their physics; this holds generally but is more obvious with the thick oil which renders *F* proportional to *v* while the light mass of the body remains unnoticed.

There is no magnetic flux yet, but a “flux,” “inductance” and “memristance” can be defined as shown further below. This may surprise some who are familiar with how the memristor device was 1971 discussed through quasi-static Maxwell theory. Let us therefore derive the non-magnetic electrical circuit theory again, this time from Maxwell’s equations, which come in two pairs. The first pair relates the electric free charge and current densities:


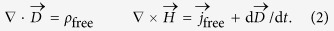


The second pair is the first pair’s magnetic twin, except for that there are no magnetic charges or currents because magnetic fields are a relativistic correction:


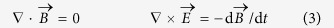


Magnetism is due to Lorentz-Fitzgerald contraction and time dilatation on moving charge distributions. The right of Eq. (2) can be written 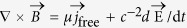. If the velocity of light *c* were much larger than it happens to be etc., we conceivably would not know magnetism. Neglecting magnetic fields is similar to concentrating on the non-relativistic limit, thus showing a more basic part of the whole theory. Similar is done in much of quantum mechanics. This is not a philosophical thought experiment imagining a world where magnetism is too weak to notice. Instead, we clarify the nature of involved symmetries by showing for example where magnetism becomes necessary. For now, with *B* and *H* negligible, only parts of Eq. (2) remain from Maxwell’s equations. Integration of *ρ*_free_ and *j*_free_ results in charge *Q* and current *I* and thus returns FR1.

### Flux, *L*, and *M* still without magnetism

Defining a “flux” *φ* = ∫*U*d*t* provides a so called second fundamental relation FR2, but unlike Eq. (6), it does *not* derive from Maxwell’s equations:


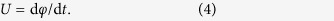


**Figure 2:**
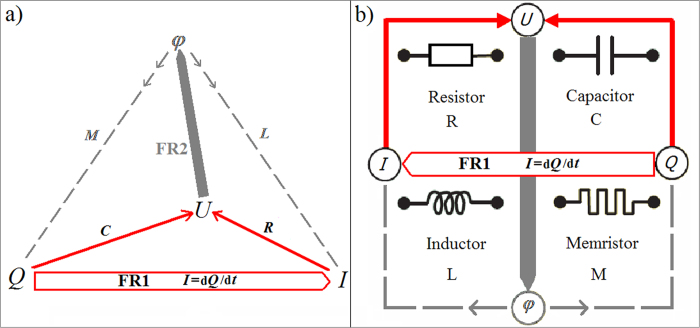
Illustration of the extended symmetry: (**a**) FR2 with the fourth variable *φ* erect a tetrahedron on top of the previous triangle. *L* and *M* label two new edges which correspond to two new BCE. (**b**) The usually given table is now complete, but without magnetism, the “inductor” cannot be the real EM inductor device.

Apart from the new *U*-to-*φ* edge (Fig. 2), there are thus yet again two more edges (almost as if we introduced another charge with a force field): *φ*-to-*I* and *φ*-to-*Q*. These correspond to two further binary relations, and thus two more BCE can be defined. *φ* is defined that way because it is thereby a canonically dual charge, because *U*, which of course relates to energy, is the dual current (compare Eq. (4), to FR1). Current is always a time derivative, and time *t* and its canonical dual, energy *E*, are the main players in dynamical physical theories. Circuit theory rests on this because energy conservation leads to Kirchhoff’s loop rule (all voltages around any closed loop in the circuit network sum to zero) and conservation of charge leads to Kirchhoff’s node rule (all currents into and out of a network node sum to zero: Σ*I* = 0). The tetrahedral construct can thus provide complete circuit theory in the sense of that the four BCE can model all potentially non-linear circuit behaviors. The two new BCE are called ideal inductor (inductance) *L*_(*φ*,*I*)_ = d*φ*/d*I* and ideal memristor (memristance)


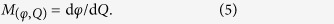


**Figure 3:**
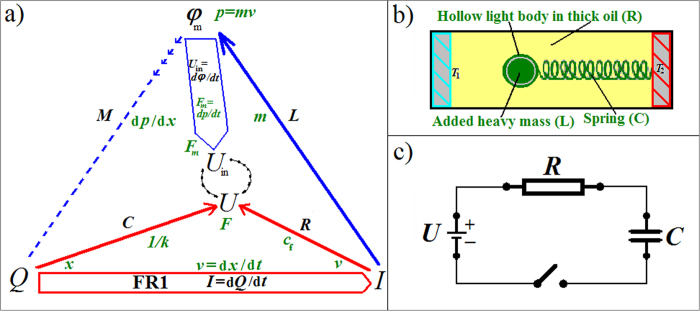
(**a**) The symmetry that suggests a real memristor device. The chains indicate that the voltages *U* and *U*_in_ are coupled by the circuit just like *U* already refers to coupled *U*_C_ and *U*_R_. (**b**) The body in viscous oil has now a heavy inertial mass *m*, therefore the BSO system can oscillate like LRC-circuits. (**c**) An ideal voltage *U* is connected parallel to an ideal capacitor *C*. When the switch closes without a resistance *R*, infinite current flows for an infinitesimal instant. *R* models the internal resistance of a voltage source. A real resistor device is not implied and may in conceivable scenarios not exist.

Because of d*φ*/d*Q* = (d*φ*/d*t*)/(d*Q*/d*t*) = d*U*/d*I*, a memristance is a resistance with standard units of [*φ/Q*] = Ohm (Ω), and a linear memristor *M* = *φ*/*Q* is just a constant Ohmic resistor. The independence between BCE therefore requires that the memristor must be in general non-linear. All the BCE are generally non-linear. Since *M* is a resistance that depends on the charge *Q*_(*t*)_, it memorizes the charge that has flown through it; hence the term “memristance.” *M* can therefore help modeling charge dependent resistors *R*_(*Q*)_, which in BSO are position dependent drag coefficients *c*_f(*x*)_. The latter can be obtained by making the oil’s density depend on *x*, for example by maintaining a temperature gradient as indicated in Fig. 1a and Fig. 3b. If it were correct that “Resistance switching memories are memristors,”^16^ we could now already claim to have found a purely mechanical memristor.

The confusing aspect is that we all know real EM inductors and thus associate them with the non-magnetic circuit theoretical “*L*”. Without magnetism, we would not suffer this confusion, and so we should first recognize that if we insist on suggesting devices, the construct suggest *two* new devices, not one. However, actually it suggests no new devices yet. The mechanical and electrical circuit theories are mathematically precise analogs by construction and one can make elaborate circuits such as the Rouse model in polymer dynamics, a chain consisting of springs and beads in a viscous fluid, which is the analog of and behaves like Lord Kelvin’s discrete LC chain model of the transatlantic telegraph cable. We described not just electrical circuit theory that has no (noticeable) magnetism but also the precisely analogous mechanical circuit theory without (noticeable) mass. Inertial mass is fundamentally inertia just like magnetism is fundamentally EM inductance, namely describing *induced* voltage (a force that would not be without the relativistic effect). So what does *L* stand for? There is no such thing yet! *L* will be something physical later, but it depends on the physics of the system and is therefore not even a single unique option, so we could go on and define other fluxes. We could switch on inertia to give the body a heavy mass *m*, or rest the BSO system in earth’s gravity field so that the light body feels buoyancy in the thick oil while its inertia remains negligible, or whatever else is conceivable.

The grounds on which the real memristor device was originally proposed is still absent. Without magnetism, there would be no real inductor device known, but this *third* device is vital to the originally predicted *missing fourth*^4^ (recall the 1971 cited similarity of Mendeleev’s work). That our magnetism, the relativistic effect, turns out to supply an EM-dual magnetic charge that behaves like the canonically dual charge is for all we know (in circuit theory anyway) a coincidence. The BSO system clarifies this.

### Refusing the non-magnetic circuit theory memristor as the missing memristor

One can now use all four BCE as the ideal mathematical constructs that they are (see also SI) and model complex circuits. However, surely nobody will claim that purely on grounds of the possible definitions and theoretical constructs we can therefore engineer real electromagnetic inductors in worlds without magnetism. The claim that other such devices are real memristor devices that were suggested on grounds of our electromagnetic inductor (and in that sense ‘corresponding’ to that EM inductor) is precisely as nonsensical because *L* and *M* are still on the precise same footing. It is not true that any odd device with features that can be conveniently modeled with *L* or *M* are already sufficient for having a real inductor device or real memristor device as intended by the 1971 proposal, because such devices were already described before and in the proposal itself. It would have made no sense to still tentatively propose that such devices *may* exist, yet the existence of a real memristor device was *hypothesized* at that point.

Differently put, assume for a moment that next to real resistors and real capacitors, researchers in a nonmagnetic world can also have real memristor devices. Precisely equivalent to how the missing memristor device was suggested, those researchers would predict a fourth, their “missing inductor.” Nevertheless, whatever devices would be discovered without magnetism, none can be the real EM inductor, but the latter is the grounds on which the original real memristor device hypothesis sits.

### With magnetism, real inductors, and the likely impossible real memristor

In order to relate to the real EM inductors that were already known as the third kind of real devices, flux must derive from the ‘magnetic pair’ of Maxwell’s equations. The electric field of Eq. (3) integrates to the magnetically *induced* voltage *U*_in_, and the integration of the magnetic field *B* results in the *magnetic* flux 
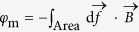
. It is also called “flux-*linkage*” because it links the magnetic field to the induced voltage. The resulting *voltage-flux (or flux-linkage)* relation relates *U*_in_ to the time derivative of the magnetic flux:


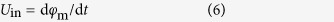


Omitting indices, it appears as if we re-derived FR2 in Eq. (4) from Maxwell’s set as done with FR1 before. As before when introducing electric force fields, which added *U* to erect a triangle, now the magnetic field adds *φ*_m_, a fourth corner that erects a tetrahedron on top of the triangle (Fig. 3a). Calculating *φ*_m_ around a Dirac monopole 
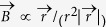
 and comparing the result *B* = *φ*_m_/(4*πr*^2^) with the electron’s *E* = *e*/(4*πr*^2^) illustrates that *φ*_m_ corresponds indeed to an EM-dual, magnetic charge.

Not the symmetry alone but it together with the real inductor corresponding to *L* may now suggest that *M* perhaps also corresponds to a real device. In the BSO system, we can switch on inertia, or if the body’s mass *m* was merely not noticeable in the thick oil, add a heavy mass *m* (Fig. 3b). The system becomes an under-damped harmonic oscillator with *m* being equivalent to an LRC circuit’s *L*^21^. *φ*_m_ = *L I* is here momentum *p* = *m v*, which happens to be again the canonical dual of the charge, here *x*. However, rather than the other way around (see arrows in Fig. 3a), these fluxes derive from the particulars of the involved physics around the known real devices (spool *L* or mass *m*), for example inertia instead of gravity. The new force d*p*/d*t* = *m a* = *F*_m_ derives from *p* like the new *U*_in_ derives from *φ*_m_ rather than the other way around. We hereby describe a new, purely mechanical ideal memristor BCE where *M* from Eq. (5) is now a non-linear d*p*/d*x* having the units of drag [*c*_f_] = kg/s. A real mechanical “inductor” device corresponds to *m*, namely the body’s heavy inert mass, from which we obtained the mechanical *M* with units kg/s in the first place whereas gravity would result in something else entirely. Given that real devices correspond to all three out of four BCE, namely a potentially very light hollow body in thick oil (R), spring (C), and the heavy mass added into the body being the third (L), we hereby hypothesize a missing fourth, namely a real mechanical memristor device (M) which needs the involvement of mass inertia just like the 1971 hypothesized memristor device requires magnetic induction. The position dependence of *c*_f(*x*)_ due to the temperature gradient is not sufficient. One could think that our mechanical memristor device is more easily found because charge *p* is a new charge with its force *F*_m_ = *m a* (inert mass is not a special relativistic effect on *x*). A mechanical memristive device (MMD) in the sense of an effective *c*_f(*x*,*v*)_ is easily constructed by a contraption that makes *m* dependent on *x*^*α*^ with a suitable power α. However, especially in the electromagnetic case one should not expect a new set of devices as if a new independent field is introduced. The symmetry would be richer if introducing new magnetic charges. Magnetic monopoles would allow magnetic capacitor devices for example. But our magnetism is a relativistic effect. Loosely speaking, it should not surprise if only half of the naively expected two new devices exist. Moreover, trying to construct a MMD with *c*_f(*x*,*v*)_ but also *m*_(*x*)_, reveals the problem with obtaining the hypothesized real memristor devices: There is no mass momentum *p* without velocity *v*. For a d*p*/d*x* independent of *v*, mass *m* must not just depend on *v* (such as in relativity). It must also depend in just the right way and yet still be inert mass with momentum even at zero velocity! The hypothesized EM memristor device may perhaps have a flux *φ*_m_ at zero current because magnetic fields can be sustained more directly as electromagnetic fields (light). This is why the hypothesized real memristor device should be expected to be found in electro-optics rather than nanotechnology.

These reasons add to previous ones^7^ for why a real memristor device is likely impossible, but we abandon all such speculative argumentations before they again distract from that our discussion of non-magnetic circuit theory already rigorously rejected the 2008 claim. This section mainly insists further on magnetism. The original proposal focused on Maxwell equations to discuss the hypothesized real memristor device and it insisted explicitly on magnetism in several places, e.g:

“… the physical mechanism characterizing a memristor device must come from the instantaneous (memoryless) interaction between the first-order electric field and the first-order magnetic field…”^1^

The 2008 claim holds magnetic flux to be irrelevant:

“The fact that the magnetic field does not play an explicit role in the mechanism of memristance is one possible reason why the phenomenon has been hidden for so long; those interested in memristive devices were searching in the wrong places. The mathematics simply require there to be a nonlinear relationship between the integrals of the current and voltage, …”^3^

This is true if focusing on their ‘perfect memristor’ and merely “the mathematics”, but we saw that it is not valid for the hypothesized real device. Others were not “searching in the wrong places” but for the actually 1971 hypothesized memristor, which has been hidden for so long because it likely does not exist. We are not swayed by an ‘argument from authority’ citing that the originator of the 1971 proposal recently claims statements such as “The memristor is characterized by a relation between the charge and the flux, defined mathematically as the time integral of the voltage, which need not have a magnetic flux interpretation”^15^ and yet worse, even “Resistance switching memories are memristors.”^16^ Such refers to the ideal memristor and other “memristors” in circuit theory, but not to the 1971 implied fourth device, even if the original ‘discoverer of the hypothesis’ may not see that clearly.

## Conclusion

We have clearly separated several usually confused issues, especially different meanings of “memristor” and “ideal/real/perfect/… memristor.” We isolated the criticizing of the claimed discovery of the originally predicted real memristor from criticizing device applications etc., and also implicitly distinguished the alternative scenarios of the original prediction being faulty in a way that could have been discovered in 1971 already versus a flawless proposal that may simply not apply to our universe (say because we have no magnetic monopoles which are perhaps necessary). The main results are: The 2008 claim is rigorously shown to be not the discovery of the “missing memristor” as it was implied by the 1971 prediction of a *fourth missing* real device, which requires a third to be already known; while merely similarly behaving systems were known already even for the memristor, the memristor hypothesis was grounded on that the real EM inductor was known to exist, which could not be known without magnetism. The 1971 prediction already confused deceptively similar looking symmetries that are fundamentally different, but we clarified the issue and saw that the original hypothesis is indeed of fundamental scientific significance and no more flawed than comparable historical predictions always are in hindsight. It demands a *magnetism requiring* real memristor device and discovering such would deserve much attention. We added further arguments for why this real memristor device does likely not exist, including a mechanical ideal memristor BCE and hypothesizing a corresponding real mechanical memristor device (in an orbiting, hollow-body-in-thick-oil system) that requires *mass inertia* just like the 1971 implied device requires *magnetic induction*; we also rejected our mechanical memristive devices and position dependent drag; they are not the missing mechanical memristor. However, rigorously speaking, the real memristor device may still be discovered, possibly in electro-optics. Accepting a false discovery is not conductive toward this aim.

A controversy developed, and in this battle, critics of the 2008 claim have been dealt harshly with. This is understandable given the confusion, because confusing issues and therefore piling up quite irrelevant criticisms is easily interpreted as bias. We criticized the critics at several points. In the interest of proper balance one should point out that the rigid defense of the 2008 claim can also appear insincere: *Either* one admits that the real memristor device has not been found *or* one attaches the label “real memristor device” more loosely, but then one must admit that such devices have been known all along. We cannot have it both ways, namely being loose with what X means while also insisting on that a very significant, fundamental scientific entity X suggested on grounds of deep insights much like the Higgs boson has been found, or to stay with the here relevant EM symmetry, Dirac’s magnetic monopole.

## Additional Information

**How to cite this article**: Vongehr, S. and Meng, X. The Missing Memristor has Not been Found. *Sci. Rep*. **5**, 11657; doi: 10.1038/srep11657 (2015).

## Supplementary Material

Supplementary Information
