# Supplementary material for: The Missing Memristor has Not been Found
Source: Sci Rep. 2015 Jun 25;5:11657. doi: 10.1038/srep11657 (PMC4479989; doi:10.1038/srep11657)
Supplement: Supplementary Information [file srep11657-s1.doc]

Electronic Supplementary Information

The Missing Memristor has Not been Found

Sascha Vongehr[[1]](#footnote-2) and Xiangkang Meng^a^

Added details may confuse especially if they involve technicalities of devices, but the following clarifications may help understanding our rigorous argument.

##

## Theoretical ideal BCE vs. real devices

BCE are *basic* elements in the following sense. **B1:** They are *independent* of each other. They span a space much like a *basis* of linearly independent vectors or axioms. One cannot connect ideal resistors together but end up with a circuit having capacitance. **B2:** They are *passive* and were called “passive circuit elements,”[[2]](#endnote-2) meaning they do not supply any energy. A battery is not a BCE. Violating B2 can violate B1. **B3:** As indicated by their symbols (Fig. 2b), they have only two terminals.

There is simplicity (“basic-ness”) involved, especially in B3, but although simple devices correspond to three of the BCE, “basic” does not mainly refer to simplicity. Also, the BCE are *theoretical ideal* entities. An ideal capacitor has no resistance at all (B1). Thus, strictly speaking, BCE are all impossible as real devices. We should not be surprised if one BCE has no corresponding, let alone simple device. Their relevance rests in modeling. An ideal *C* parallel to an ideal source *U* (Fig. 3c) results in infinite current *I* (for an infinitesimal instant). Hence, there must be a resistance *R*, but it is not a device we need to wire up. We put *R* into circuit diagrams and equations. It usually models the internal resistance of the source. The ideal memristor *M* helps modeling systems whose behavior cannot be modeled with help of only *L*, *R*, and *C*. Modeling with charge dependent resistances can be complicated. Tracking memristive devices in $U\times I$ space, there will be resistant switching, pinched hysteretic loops, hysteretic conductance as well as apparent negative differential resistance. The significance of the ideal memristor rests in circuit theoretical modeling which could be conceivably important in a world without magnetism, but for this very reason, it has not got the further relevance of relating to fundamental symmetries in a way that predicts yet undiscovered systems. The latter made the suggested real memristor device so intriguing, because it corresponds to the EM inductor as a sort of complementary EM-symmetry counterpart, much like electrons suggest magnetic monopoles.

## Memoryless memory

*M*(*_Q_*) memorizes the charge that has flown. However, the first quote in the main text

“… the physical mechanism characterizing a memristor device must come from the instantaneous (memoryless) interaction between the first-order electric field and the first-order magnetic field…”[[3]](#endnote-3)

demands “instantaneous (memoryless) interaction” in the hypothesized device. There should be no material memory such as in the devices submitted with the 2008 claim, where the current through the thin film consists partially of ionic charge carriers such as oxygen vacancies. These enter the film (e.g. TiO_2_) when starting from the high resistance state. They thus increasingly dope the film with such easily charged impurities, which lowers the resistance to electrons. In other systems, induced metallic precipitates may lower the overall resistance. The width *w* of the low resistance region grows and the resistance of the film therefore decreases. No instantaneously interacting EM fields are involved. The doped region is the material based memory which remains if the device is disconnected. This is not the “memoryless” memristor originally postulated.

The thin films are not the sought missing memristor also because they can be modeled by memristance only as long as *w* is between zero and the nano width *W* of the film, which cannot be thicker because of the dynamic instability (not to mention a thermodynamic long term instability) of the doped region’s moving boundary shape and because the memristance property vanishes with *W*. (Both aspects are unavoidable[[4]](#endnote-4) as they do not depend on the material. For example, memristance is undetectable in thick films because the time *t* over which the resistance changes is proportional to *W*^2^.) Whenever *w* approaches 0 or *W*, the current must be reversed. The original memristor proposal demanded an alternate-current device to ensure the “instantaneous (memoryless) interaction” of EM-fields, not because material memory can be exhausted. That the correspondence to a BCE is only satisfied for short times should not be acceptable and is not accepted in case of other BCE. Capacitors supply energy when discharging and would not be passive (B2) if we were allowed to observe them for select, short durations in order to categorize the device. Batteries, which are a fitting comparison considering the redox-reactions in the thin films, can briefly seem like de-charging capacitors but are not BCE.

**References**

1. Corresponding authors’ electronic mail: vongehr@usc.edu mengxk@nju.edu.cn [↑](#footnote-ref-2)
2. Feynman, R., Leighton, R. B. & Sands, M. L. The Feynman lectures on physics vol. 1. Pearson Education Inc. chapter 23-3, page 23-5 to 23-6, (1965). [↑](#endnote-ref-2)
3. Chua, L. O. Memristor – the missing circuit element. *IEEE Trans. On Circuit Theory* **CT-18**, 507-519 (1971). [↑](#endnote-ref-3)
4. Vongehr, S. Missing the Memristor. *Adv. Sci. Lett.* **17**, 285-290 (2012). [↑](#endnote-ref-4)
